# Periodontitis pathogen *Porphyromonas gingivalis* promotes chronic obstructive pulmonary disease via affecting neutrophils chemotaxis and function

**DOI:** 10.1038/s41368-025-00397-1

**Published:** 2026-01-09

**Authors:** Luyao Zhang, Huan Tian, Yuanyuan Ma, Jing Xu, Chang Guo, Zuomin Wang, Jie Ma

**Affiliations:** 1 Center of Biotherapy, Beijing Hospital, National Center of Gerontology, Institute of Geriatric Medicine, Chinese Academy of Medical Sciences, Beijing, China; 2 Changsha Stomatological Hospital, Hunan University of Traditional Chinese Medicine, Changsha, China; 3 Department of Stomatology, Beijing Chaoyang Hospital, Capital Medical University, Beijing, China; 4 Department of Clinical Laboratory Diagnostics, Beijing Friendship Hospital, Capital Medical University, Beijing, China; 5 Medical School, University of Chinese Academy of Sciences, Beijing, China

**Keywords:** Periodontitis, Experimental models of disease, Molecular medicine

## Abstract

Chronic obstructive pulmonary disease (COPD), a disease responsible for early mortality worldwide, is well accepted to be associated with periodontitis epidemiologically. Although both of the diseases are the multi-microbial inflammatory disease, the precise underlying mechanisms by which periodontitis influences the progression of COPD remains largely unknown. Here, we established COPD accompanied with periodontitis mouse models and observed the pronounced progress in pulmonary symptoms and histopathology, characterized by poorer respiratory function, thickened bronchial walls, and increased neutrophils infiltration in lung tissue. Mechanistically, periodontitis pathogen *Porphyromonas gingivalis* (*P. gingivalis*) relocated in the lung through the respiratory tract and LPS from *P. gingivalis* promoted the secretion of chemokines CXCL2 and G-CSF of alveolar epithelial cells through NF-κB and p38 MAPK pathways to recruit neutrophils. Furthermore, exposure to *P. gingivalis* of infiltrated neutrophils released matrix metallopeptidase-8 (MMP-8) and neutrophil elastase (NE), which aggravated airway inflammation and tissue damage. These findings indicated that periodontitis could exacerbate COPD via its pathogen *P. gingivalis*, which translocated in the lung and stimulated neutrophil chemotaxis and activation in the lung.

## Introduction

Chronic obstructive pulmonary diseases (COPD) is a highly prevalent chronic airway inflammatory disease characterized by an irreversible loss of lung function and persistent airflow restriction.^1^ Several epidemiological and clinical studies have affirmed the correlation between periodontitis and COPD,^2–4^ and periodontitis has been further identified as an elevated risk factor for COPD development.^5,6^ The increased risk of mortality for COPD is also significantly associated with periodontitis severity.^7^ Moreover, common risk factors shared by periodontal disease and COPD, such as smoking, age and economic factors, further provide support for the relevance of COPD and periodontitis.^8^ However, there is currently a lack of clues regarding the direct link between periodontitis and COPD.

Periodontitis is a common oral chronic inflammation caused by dental plaque, calculus and other local factors, which is characterized by the destruction of alveolar bone and the alteration of the oral microbiota.^9,10^ Poor dietary habits and oral hygiene disrupt the equilibrium of the oral microbial ecosystem, affecting the occurrence and progression of periodontitis.^11^ Oral microbiota and its virulence factors can invade periodontal tissue and subsequently enter the blood circulation, leading to several systemic diseases, such as diabetes,^12^ cardiovascular diseases,^13^ Alzheimer’s disease.^14^ Our previous research reported that *Porphyromonas gingivalis (P. gingivalis)*, the most important gram-negative pathogen of periodontitis,^15,16^ can enter pancreas through the digestive tract, remodel the inflammatory microenvironment, and promote the development of pancreatic cancer.^17^ Notably, since the lungs and lower respiratory tract are linked to the upper respiratory tract and oral cavity, whether *P. gingivalis* can colonize the lungs through the respiratory tract and alter the pulmonary microenvironment affecting the progression of COPD deserves further investigation.

For COPD patients with impaired ability of bacteria clearance, recurrent infections exacerbate the decline in lung function.^18^ To defense against bacterial pathogens, neutrophil acts as the first line of innate immune response.^19^ However, neutrophils are also implicated in the development of alveolar destruction in COPD through releasing destructive mediators such as neutrophil elastase (NE) and matrix metalloproteinases (MMPs).^20^ Thus, the level of airway neutrophilic inflammation is associated with disease severity and exacerbations of COPD.^21^ Nevertheless, the precise underlying mechanism that periodontitis potentially exacerbated COPD through the interaction of crucial periodontitis pathogen and neutrophils remains to be fully elucidated.

In this study, we focused on the effect of *P. gingivalis* on lung microenvironment to explore the impact of periodontitis on the progression of COPD. Establishing a mouse COPD model, we found the presence of periodontitis could accelerate the development of COPD through its pathogen *P. gingivalis* resulting in lung malfunction. Lipopolysaccharide (LPS) derived from *P. gingivalis* activates the NF-κB and p38 MAPK pathways in alveolar epithelial cells to produce chemokines Granulocyte Colony-Stimulating Factor (G-CSF) and C-X-C Motif Chemokine Ligand 2 (CXCL2), which induced the accumulation of neutrophils. Further investigation disclosed the key role of neutrophils in *P. gingivalis* mediated inflammation in the lung, which markedly induced the condition aggravation in COPD. Altogether, our data indicated that periodontitis associated pathogen *P. gingivalis* plays an important role in COPD progression, providing a new therapeutic strategy for COPD patients with periodontitis. In the clinical diagnosis and treatment of COPD patients, the matter of oral hygiene should be taken into account. The proper treatment of periodontal diseases has the potential to alleviate the disease progression of COPD.

## Results

### Periodontitis associated pathogen *P. gingivalis* colonizes the lung through the respiratory tract

**Fig. 1:**
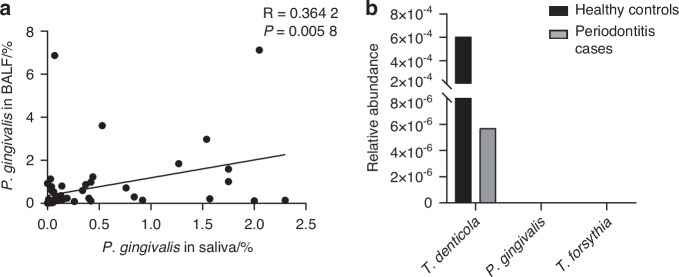
Periodontitis associated pathogen *P. gingivalis* colonizes the lung through the respiratory tract. **a** Correlation scatter plots of the proportion of *P. gingivalis* in saliva with the proportion of *P. gingivalis* in paired bronchoalveolar lavage fluid (BALF) (*n* = 56). This analytical data was derived from the public database PRJNA757607. The *p* and *R* values were calculated by Spearman correlation analysis. **b** The relative abundance of periodontitis associated pathogens in the blood of periodontitis patients (*n* = 17) and healthy controls (*n* = 19). This analytical data was derived from the public database PRJNA659735

Due to the discovery of species with highly similar 16S rDNA sequences to oral periodontitis associated pathogens in the trachea, including *P. gingival*is,^22^ we first investigated the correlation between *P. gingival*is in the lung and oral cavity. The analysis of the metagenomic database of paired human saliva and bronchoalveolar lavage fluid (BALF) samples revealed a positive correlation between the content of *P. gingivalis* in the lung and oral cavity (Fig. 1a), supporting that the oral cavity could be a reservoir for lung *P. gingivalis*. We further analyzed the blood microbiome database and found that no detectable *P. gingivalis* in the blood microbiome sequencing of either periodontitis patients or healthy controls (Fig. 1b), suggesting that *P. gingivalis* in the lung is mainly derived from direct inhalation rather than blood circulation. These results indicated periodontitis associated pathogen *P. gingival* colonizes the lung through the respiratory tract.

### Periodontitis associated pathogen *P. gingivalis* promotes COPD progression

**Fig. 2:**
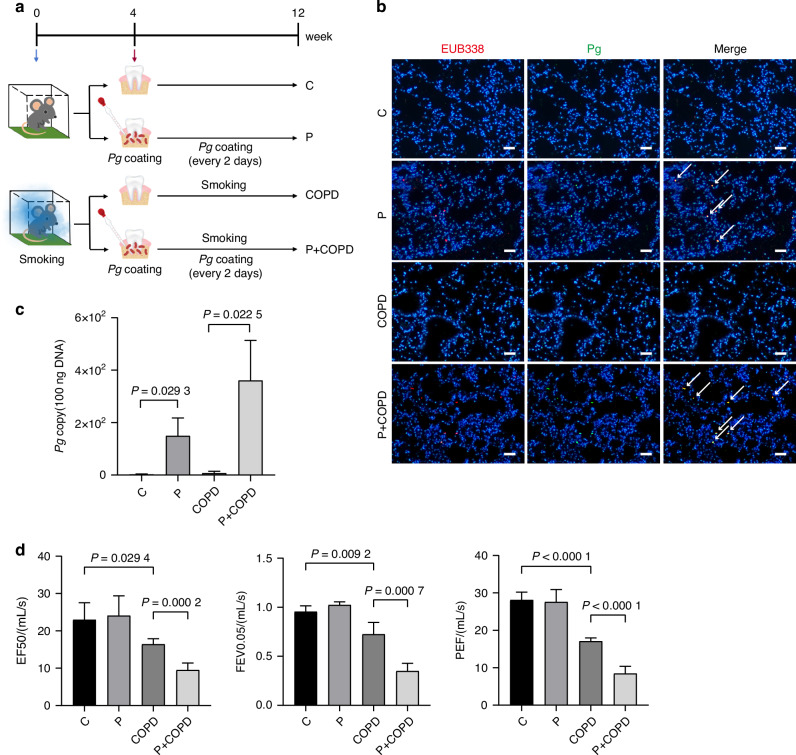
Periodontitis associated pathogen *P. gingivalis* promotes COPD progression. **a** Schematic diagram showing construction of periodontitis and COPD to determine the effect of periodontitis on COPD progression. **b** Representative fluorescence in situ hybridization (FISH) images of *P. gingivalis* in lung tissues of mouse with control (C group), periodontitis (P group), COPD induced by cigarette smoking (COPD group) and COPD combined with periodontitis (P + COPD group). Blue: DAPI-labeled nuclei; red: Cy3-EUB338, a universal bacterial 16s rRNA-directed oligonucleotide probe, green: Alexa 488- PGOI, a *P. gingivalis*-specific probe. Scale bars: 50 µm. **c** *P. gingivalis* 16S DNA gene copy number in lung tissue of each group was quantified by TaqMan qPCR (*n* = 5 per group). **d** Lung function test results in each group (*n* = 6 per group). Significant differences were assessed using one-way ANOVA

We recognized that mice with periodontitis showed the pathological state of pulmonary inflammation with thickening of blood vessel wall, bronchial wall and obvious neutrophils infiltration.^23^ Therefore, to assess whether periodontal pathogens is an aggravating risk factor for the progression of COPD, we generated mouse models categorized into four groups: control (C group), periodontitis (P group), COPD induced by cigarette smoking (COPD group) and COPD combined with periodontitis (P + COPD group) (Fig. 2a). Initially, we examined the construction of periodontitis by H&E observation and found significant alveolar bone destruction in mice treated with *P. gingivalis* (Supplementary Fig. 1a, b). The oral microbiome easily enters the respiratory system, which is the prerequisite that periodontal diseases affect lung diseases. To further confirm the presence of *P. gingivalis* in the lung of mice, we performed ribosomal RNA (rRNA) fluorescence in situ hybridization (FISH) using universal bacterial probe EUB338 and *P. gingivalis* oligonucleotide probe PGOI. *P. gingivalis* FISH plaques were detected in mice with periodontitis (Fig. 2b). Additionally, DNA content of *P. gingivalis* was more abundant in both P group and P + COPD group (Fig. 2c). Given that Lipopolysaccharide (LPS) is an important virulence factor for *P. gingivalis*,^24^ we further detected LPS by immunohistochemistry (IHC) and found that LPS expression was significantly higher in both P and P + COPD groups (Supplementary Fig. 1c, d). These results further confirmed that periodontitis-associated pathogen *P. gingivalis* indeed accessed to the lung.

Subsequently, we evaluated the impact of P. *gingivalis*-induced periodontitis on COPD. H&E staining of lung tissue suggested that both P + COPD and COPD groups exhibited pronounced dilated and fractured alveolar walls (Supplementary Fig. 2a, b). Compared with the COPD group, the presence of P. *gingivalis* in the P + COPD group significantly associated with increased thickness of bronchial walls, and inflammatory cells exudation were observed in alveolar space (Supplementary Fig. 2a, b). Correspondingly, in contrast to the respective control C and COPD groups, increasing trend of the protein levels of inflammatory cytokines IL-1β, TNF-α were detected in the lung tissues of P and P + COPD groups (Supplementary Fig. 2c). Moreover, mice in P + COPD group showed increased inflammatory cells in bronchial alveolar lavage fluid (Supplementary Fig. 2d, e). Notably, Lung function test results revealed that the P + COPD group had decreased EF50 value, FEV0.05 value and PEF value as compared to those of COPD group (Fig. 2d). These results indicated that the presence of periodontitis associated *P. gingivalis* induced pulmonary inflammation, worsened lung function and facilitated the progress of COPD.

### *P. gingivalis* promotes recruitment of neutrophils in the lung which produce inflammatory cytokines

**Fig. 3:**
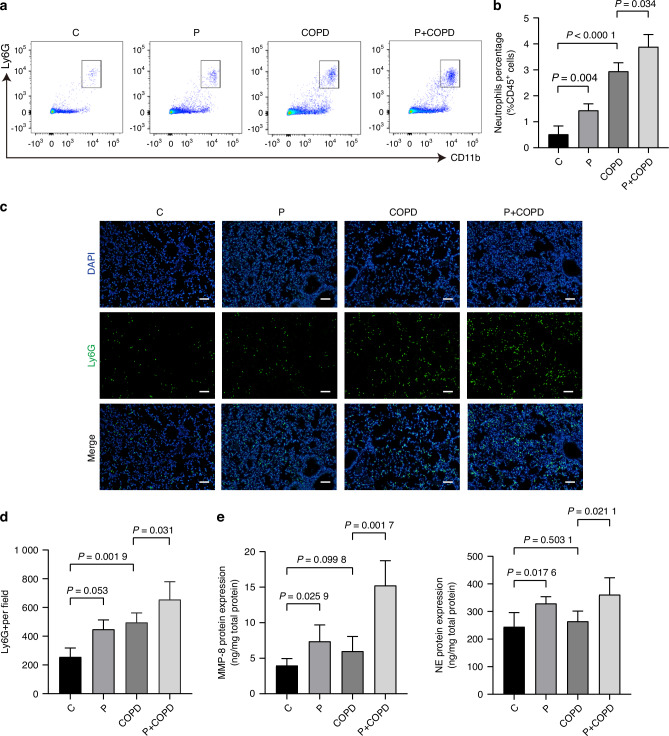
*P. gingivalis* promotes recruitment of neutrophils in the lung which produce inflammatory cytokines. **a** Representative plots showing neutrophil level in lung of each group by flow cytometry. **b** Quantitative analysis of neutrophil level in lung in (**a**) (*n* = 6 per group). **c** Representative immunofluorescence images of Ly6G positive cells in lung tissue of each group. Scale bars: 100 µm. **d** Quantitative analysis of Ly6G positive cell in (**c**) (*n* = 4 per group). **e** The protein expression and secretion of matrix metalloproteinases (MMP)-8 and neutrophil elastase (NE) in lung tissue of each group was measured by ELISA assay (*n* = 6 per group). Significant differences were assessed using one-way ANOVA

To further determine the mechanism by which periodontitis aggravates COPD, we investigated the immune microenvironmental changes in the lung of mice when periodontitis occurred. Given that activated neutrophils are the major pathogen-fighting immune cells closely associated with the progression of COPD,^25,26^ we evaluated the change of neutrophil level in the lung tissue and peripheral blood. Flow Cytometry results revealed that neutrophils were enriched in the COPD groups, and the increase of neutrophils was more pronounced in the P + COPD group than COPD group in both lung tissues and peripheral blood (Fig. 3a–d and Supplementary Fig. 3a, b).

Neutrophils in activate state produce several proteinases and oxidative enzymes which can degrade extracellular matrix components.^27^ In this study, we observed high expression of MMP-8 and neutrophil elastase (NE) in P + COPD group in both mRNA and protein level, while no significant expression in COPD group although there was a large number of neutrophils in the lung (Supplementary Fig. 3c and Fig. 3e). Considering the difference between the groups was the existence of *P. gingivalis* in the lung, we then hypothesized that the expression of MMP-8 and NE by neutrophils might be influenced by *P. gingivalis* infection. This hypothesis was proofed by an in vitro experiment which showed that *P. gingivali*s promoted the production of MMP-8 and NE by neutrophils isolated from normal peripheral blood (Supplementary Fig. 3d). Altogether, these results demonstrated that periodontal pathogen *P. gingivalis* could induce neutrophils accumulation and proteinases release in the lung, which were important contributors to the aggravation of COPD.

### LPS promoted alveolar epithelial cells to produce neutrophil attractants through NF-κB and p38 MAPK pathway activation

**Fig. 4:**
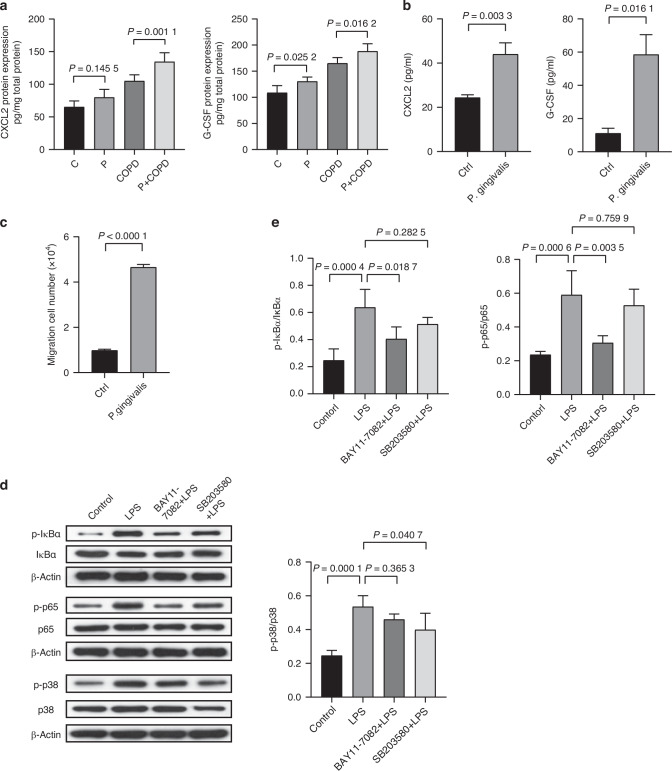
LPS promoted alveolar epithelial cells to produce neutrophil attractants through NF-κB and p38 MAPK pathway activation. **a** The protein expression and secretion of C-X-C Motif Chemokine Ligand 2 (CXCL2) and Granulocyte Colony-Stimulating Factor (G-CSF) in lung tissue of each group was measured by ELISA assay (*n* = 6 per group). **b** The protein expression and secretion of CXCL2 and G-CSF in MLE-12 cells treated with *P. gingivalis* [MOI] = 1:100 for 24 h (*n* = 3 per group). **c** The analysis of neutrophils chemotaxis assay. **d**, **e** MLE-12 cells were pre-treated with 10 μmol/L BAY11-7082 (NF-κB inhibitor) or 20 μmol/L SB203580 (p38MAPK inhibitor) for 2 h and then treated with 1 μg/mL LPS for 24 h. inhibits activation by LPS and inhibits activation by LPS. **d** Representative images of p-IκBα, p-p65 and p-p38 western blotting bands; **e** Quantitative analysis of Western blotting in (**d**) (*n* = 3 per group). Significant differences were assessed using two-tailed unpaired Student’s *t* test and one-way ANOVA

We further explored the molecular determinants responsible for *P. gingivalis* associated neutrophil infiltration. First, we observed upregulated protein levels of C-X-C Motif Chemokine Ligand 2 (CXCL2) and Granulocyte Colony-Stimulating Factor (G-CSF), which are recognized as neutrophil attractants, in the lung tissue of P + COPD group (Fig. 4a). In order to disclose whether this elevation was associated with *P. gingivalis*, mouse alveolar epithelial cells MLE-12 was cultured in vitro and stimulated with *P. gingivalis* at 100 multiplicity of infection (MOI) for 24 h. The expression levels of neutrophil chemokines CXCL2 and G-CSF in MLE-12 were then detected. The results showed that viable *P. gingivalis* markedly increased the expression and secretion of neutrophil chemokines in alveolar epithelial cells (Supplementary Fig. 4a and Fig. 4b) in vitro. Moreover, we performed neutrophil chemotaxis assay in this system stimulated by *P. gingivalis*, and found that the supernatant of alveolar epithelial cells (AECs) stimulated by *P. gingivalis* could recruit a greater number of neutrophils (Fig. 4c). These results indicated that alveolar epithelial cells recruited neutrophils under stimulation of *P. gingivali*s.

We further investigated the influence of LPS derived from *P. gingivalis* on alveolar epithelial cells since it was reported that LPS, an important virulence factor for *P. gingivalis*, can cause lung injury by inducing inflammation and apoptosis of alveolar epithelial cells.^28,29^ We evaluated mRNA expression of CXCL2 and G-CSF in MLE-12 cells exposed to different doses of LPS from *P. gingivali*s. The results showed that LPS observably upregulated the expression of these two chemokines in MLE-12 cells in a concentration-dependent manner (Supplementary Fig. 4b). At the protein level, the secretion of CXCL2 and G-CSF from MLE-12 cells were also significantly increased under the stimulation of LPS (Supplementary Fig. 4c).

Previous studies have shown that the expression of CXCL2 and G-CSF is regulated by p38 MAPK and NF-κB signaling pathways.^30,31^ Thus, we next investigated the molecular mechanisms of LPS in inducing the expression of CXCL2 and G-CSF and found that both NF-κB (IκBα and p65 phosphorylation) and p38 MAPK (p38 phosphorylation) pathways were activated by LPS from *P. gingivali*s (Fig. 4d, e). Importantly, the pathway activation by LPS could be significantly attenuated by BAY11-7082 (NF-κB inhibitor) and SB203580 (p38 MAPK inhibitor), respectively (Fig. 4d, e). Furthermore, we found that both inhibitors significantly reduced the levels of secreted CXCL2 and G-CSF (Supplementary Fig. 4d), suggesting that LPS induced chemokine secretion from alveolar epithelial cells via NF-κB and p38 MAPK pathways.

### Inhibiting neutrophil chemotaxis could alleviate COPD progression

**Fig. 5:**
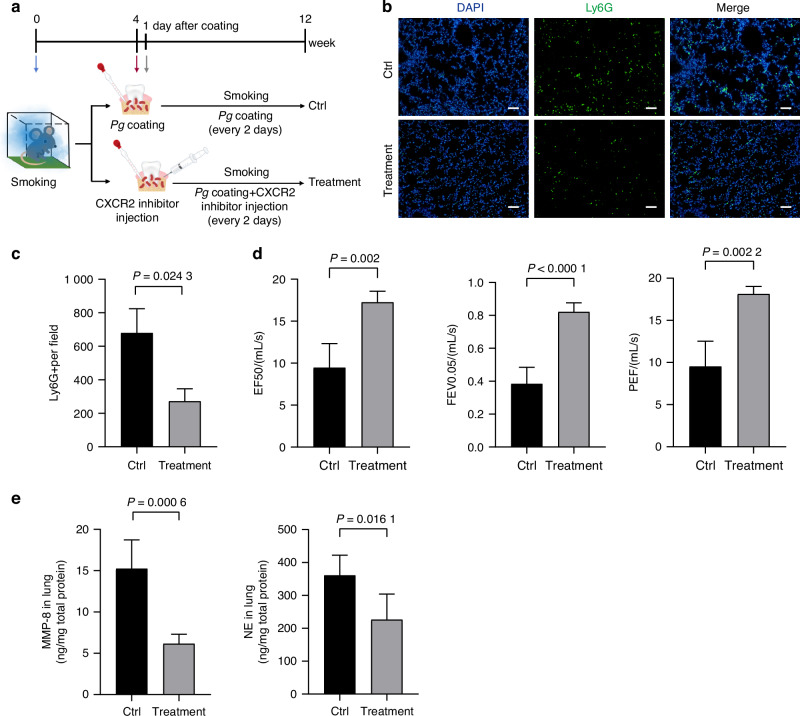
Inhibiting neutrophil chemotaxis could alleviate COPD progression. **a** Schematic diagram of CXCR2 antagonist administration to C57BL/6 mice with COPD combined with periodontitis. **b** Representative immunofluorescence images of Ly6G positive cells in lung tissue of each group. **c** Quantitative analysis of Ly6G positive cell in (**b**) (*n* = 4 per group). **d** Lung function test results in mouse with COPD combined with periodontitis (Ctrl), and those treated with CXCR2 antagonist (Treatment) (*n* = 5 per group). **e** The protein expression and secretion of MMP-8 and NE in lung tissue of each group was measured by ELISA assay (*n* = 4 per group). Significant differences were assessed using two-tailed unpaired Student’s *t* test

To further investigate the essential function of neutrophils in the exacerbation of COPD, we blocked the recruitment of neutrophils in COPD combined with periodontitis model (Ctrl) using CXCL2 receptor CXCR2 antagonist (Treatment) (Fig. 5a). First, we found that CXCR2 antagonist treatment effectively reduced neutrophil infiltration in the lung of mice (Fig. 5b, c), which significantly improved the lung function (Fig. 5d). Whereas, the expression of LPS detected by IHC was not affected by the blockade of neutrophil chemotactic (Supplementary Fig. 5a, b). We also evaluated lung tissue destruction by H&E staining and found that chemokine blockade markedly improved the proportion of alveolar space area, mean linear intercept of alveoli and thickness of bronchial walls (Supplementary Fig. 5c, d). The inflammatory exudation in alveolar space was alleviated with the blockade.

MMP-8 and NE, the destructive proteinases produced by neutrophils, were significantly inhibited in the lung of COPD after chemokine blockade (Fig. 5e). In addition, the protein levels of proinflammatory cytokines IL-1β and TNF-α exhibited decreasing trend following CXCR2 antagonist treatment (Supplementary Fig. 5e). Altogether, these results revealed that *P. gingivalis-*induced neutrophil chemotaxis by alveolar epithelial cells had a great contribution to inflammation development in COPD.

## Discussion

Forecasting analysis for the Global Burden of Disease revealed that COPD would become one of the four leading global causes of disability-adjusted life-years in 2050.^32^ Therefore, it is wise to seek intervention scenarios to meet the challenge of the disease so that to reduce the upcoming health burden worldwide. The fact that the cause and mechanism of COPD is still not clear enough leads to difficulties to the disease prevention and treatment. In this study, we managed to disclose the mechanical link between periodontitis and COPD, two common chronic inflammatory diseases related to increased life expectancy.

Growing evidences show patients with periodontitis are more susceptible to COPD than those without periodontitis, and vice versa.^33^ Epidemiological findings exhibit an increased risk of COPD associated with poor periodontal health, no regular dental care, and lack of oral health knowledge.^34^ Moreover, periodontal treatment in COPD patients afflicted with chronic periodontitis can improve lung function and decrease the severity of COPD symptoms,^35,36^ suggesting that oral interventional approaches may have a beneficial impact on improvement of pulmonary prognosis. Although the above facts evidenced the probable association between the two diseases, the exact mechanism by which periodontitis affects COPD progression is not fully understood and remains to be investigated.

Periodontitis is a chronic inflammatory disease caused by bacterial biofilms. Among periodontal pathogens, *P. gingivalis* is a key candidate in the relationship between periodontitis and respiratory diseases.^37^ For COPD, significant negative correlations were found between *P. gingivalis* and FEV1%, indicating that the relative content of *P. gingivalis* in the oral cavity can deteriorate the lung function.^38^ Therefore, to investigate the promotion ability of periodontitis on COPD, we constructed mouse periodontitis models by combining ligation of mouse molars with oral infection with *P. gingivalis* strain W83. The exacerbating effect of periodontitis on COPD development was observed in our mouse model, characterized by the reduction in respiratory function and pronounced dilated and fractured alveolar walls. Importantly, in the absence of pathogenic microbes, that is to say the periodontitis mouse model induced solely by silk ligature, the lung microenvironment did not show significant change, further emphasizing the crucial role of *P. gingivalis*.

Oral microbes with specific virulence factors can invade periodontal tissues and subsequently enter the systemic circulation.^39^ Whereas, by analyzing the blood bacterial database,^40^ we found that there were no detectable *P. gingivalis* in the circulation of periodontitis patients (Fig. 1b), suggesting that *P. gingivalis* in the lung is mainly derived from direct inhalation. In our periodontitis model, we observed the accumulation of pathogen *P. gingivalis* in the lung, and this accumulation of bacteria through air tract could be accelerated accompanying the development of COPD (Fig. 2b, c).

*P.gingivalis* influences the lung microenvironment by two ways, one is body compartments of the bacteria, the other one is metabolites. LPS is an outer membrane component of Gram-negative bacteria, which induces secretion of proinflammatory mediators in invaded tissues, including cytokines, chemokines and interleukins.^41^ A previous study has reported that LPS can cause lung injury and induce the release of inflammatory signals from lung tissue and epithelial cells, including tumor necrosis factor-α (TNF-α), interleukin-1β (IL-1β), monocyte chemoattractant activating protein-1 (MCP-1) and zonula occludens (ZO-1).^42^ In addition, LPS can promote M1 macrophage polarization, secrete proinflammatory cytokines such as TNF-α and IL-1β, aggravate lung inflammatory response in the early stage.^43^ In our system, neutrophils chemoattractants G-CSF and CXCL2 significantly increased in LPS-stimulated MLE-12 cells in a concentration-dependent manner, which resulted in the recruitment of neutrophils in the lung. Given that LPS-induced activation of p38 MAPK and nuclear NF-κB is typically involved in the secretion of pro-inflammatory cytokines and chemokines,^44^ BAY11-7082 (NF-κB inhibitor) and SB203580 (p38 MAPK inhibitor) were applied to block related pathways. The results showed that both inhibitors could significantly inhibit the secretion of G-CSF and CXCL2, indicating that the p38 MAPK and NF-κB pathways play a key role in modulating LPS-induced cytokine production in our system.

Neutrophils, known as the first line of immune defense against bacterial infection, noticeably increased in the lung in mice with *P. gingivalis-*induced periodontitis. Due to the association between neutrophil infiltration and the severity and progression of COPD disease,^45,46^ we investigated the contribution of periodontitis induced accumulation of neutrophils in the lung to COPD promotion. The application of an antagonist targeting the critical ligand CXCR2 to block neutrophils recruitment reversed the progression of COPD aggravated by periodontitis, indicating the promotion of COPD by periodontal pathogens mainly through neutrophils. Neutrophils can secrete serine proteases MMP-8 and NE in the lung, leading to alveolar destruction (Fig. 3e).^47^

**Fig. 6:**
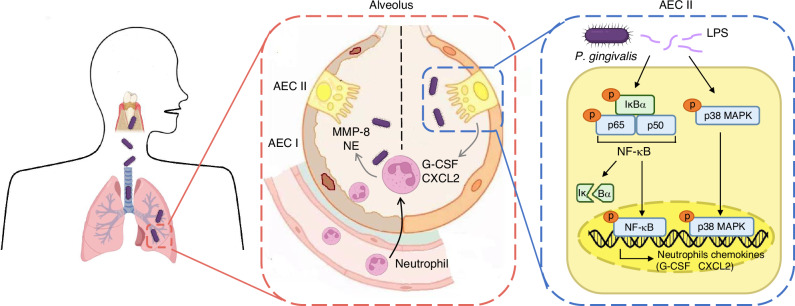
Schema depicting the contribution of *P. gingivalis* in the development process of COPD. Periodontitis associated pathogen *P. gingivalis* in oral cavity enter the lung through the respiratory tract, and its LPS can activate the NF-κB and p38 MAPK pathway in alveolar epithelial cells to produce CXCL2 and G-CSF, and then recruit neutrophils to secrete alveolar destructive mediators MMP-8 and NE to facilitate the exacerbation of COPD

In summary, we discovered that *P. gingivalis* of periodontitis and precisely the LPS from *P. gingivalis* were aggravating factors for COPD in animal models. Compared to COPD mice without periodontitis, increased expansion and destruction of pulmonary alveoli and significantly decreased lung function parameters were observed in COPD mice with periodontitis. Further mechanical study revealed that periodontitis associated pathogen *P. gingivalis* in oral cavity could enter the lung through the respiratory tract, where its principal virulence factor LPS activated the NF-κB and p38 MAPK pathways in alveolar epithelial cells. The phosphorylation of both pathways resulted in CXCL2 and G-CSF production, which recruited neutrophils to the lung to secret destructive mediators in response to LPS to facilitate the exacerbation of COPD (Fig. 6).

The limitation of the study is that we did not explore other immune cells beyond neutrophils which might be involved in the promoting effect of *P. gingivalis* in COPD. Studies have reported that periodontal pathogens can stimulate the polarization of macrophage towards M1 to promote the expression of TNF-α and IL-1β, and activate Th17 or γδT lymphocytes to release IL-17.^48–50^ These cytokines might facilitate the recruitment of more neutrophils in the lung, exacerbating airway inflammation in COPD and the disruption of alveolar cells.^51^ Additionally, our study elucidated the mechanism of *P. gingivalis*, one of the major pathogens in periodontitis, inducing pulmonary inflammation to promote COPD, while the mechanism of other periodontitis related pathogens (such as *T. denticola*) on COPD needs further study. Moreover, smoking induction was used in the establishment of our experimental COPD model, but smoking is also an important contributing factor to periodontitis, which may potentially affect the interaction between COPD and periodontitis.^52^ Therefore, we will explore more appropriate experimental modeling methods in future studies.

A number of cohort studies have indicated that periodontitis is an independent risk factor for COPD. These studies suggested that improving oral hygiene habits (such as regular tooth brushing and flossing) in patients, or conducting necessary periodontal therapy to reduce the inhalation of *P. gingivalis*, may lower the frequency of acute exacerbations of COPD.^3,53^ However, more large-scale clinical trials are still required for validation. Our subsequent research will be concentrated on the signaling pathways of other immune cells and the functions of other oral pathogens in periodontitis promoting COPD, in the hope of offering more comprehensive cues for relevant clinical intervention studies.

## Materials and methods

### Analysis of the microbiome in clinical samples

(1) The data source of saliva and paired bronchoalveolar lavage fluid (BALF) microbiome was from the NCBI SRA public database (PRJNA757607).^54^ This study enrolled 56 participants (including 33 patients with lung cancer and 23 healthy volunteers without lung disease). The proportion of males was 45%, the average age was 50.6, and the proportion of those with smoking history was 30%. For quality control and removal of host contamination, Trimmomatic was run with the following default arguments: “SLIDINGWINDOW:4:20 MINLEN:70”.^55^ The minimum length was computed as 70% of the input read length. GRCH38 was used as the host reference genome.^56^ Kraken2 (v2.0.7) was used to align the reads from each sample against the constructed database.^57^ A nonparametric Spearman’s correlation test was used to test the associations between continuous variables. (2) The data source of blood microbiome was from the NCBI SRA public database (PRJNA659735).^40^ This study enrolled 17 periodontitis patients and 19 healthy controls. Periodontitis participants were diagnosed with generalized active periodontitis via Basic Periodontal Examination (BPE). The proportion of males was 23.5%, the average age was 47, no smoking history. Control participants demonstrated pristine periodontal health. The proportion of males was 21%, the average age was 40, no smoking history. For quality control, Paired-end reads (PE) were subjected to quality filtering with specified parameters^58^ using the Qiime (V1.7.0)^59^ quality control process.

### *P. gingivalis* culture

*P. gingivalis* strain (W83) was purchased from ATCC and grown in brain heart infusion broth (BHI) (Solarbio, Beijing, China) medium containing vitamin K (1 μg/mL) and hemin (5 μg/mL) under anaerobic conditions for 24–48 h. For mice oral infection, it was centrifuged at 1 459 × *g* for 10 min at room temperature. The supernatant was discarded, and the precipitate was washed three times with phosphate-buffered saline (PBS), then the liquid medium was resuspended in a 2% carboxymethylcellulose (CMC) solution. The concentration of bacteria in the suspension was 1 × 10^9^ colony-forming units (CFU).

### Cell lines culture

Murine pulmonary epithelial cell line MLE-12 was purchased from the ATCC and maintained in high-glucose Dulbecco’s modified Eagle’s medium (DMEM, HyClone, USA) containing 10% fetal bovine serum (FBS, Gibco, USA) in a 5% CO_2_ humidified atmosphere at 37 °C. LPS-PG (Lipopolysaccharide from *P. gingivalis*) was purchased from InvivoGen (USA) and configured with PBS as a 5 mg/mL storage solution. BAY11-7082 (NF-κB inhibitor) and SB203580 (p38 MAPK inhibitor) were purchased from APExBIO (USA) and configured with DMSO for 40 mmol/L and 10 mmol/L storage solution respectively. MLE-12 were stimulated with *P. gingivalis* at 100 multiplicity of infection (MOI) for 24 h.

### Experimental mouse model

Specific pathogen free (SPF) C57BL/6J mice (Male, 6-week-old) were purchased from Beijing Huafukang Bioscience Co. Inc. (China). The method for inducing the mouse model of periodontitis was presented as follows. Firstly, the bilateral maxillary second molars of mice were ligated with 5.0 silk sutures. One day after ligation, *P. gingivalis* (10^9^ CFU/mL, 0.2 mL per mice) was applied to the buccal and lingual sides of the ligature silk in the maxilla once every 2 days and this procedure was sustained for 8 weeks. To evaluate the effects of periodontitis on COPD, COPD model was established by exposing mice to mainstream cigarette smoke (CS) through a tobacco smoke inhalation experiment system for small animals (Sibata Scientific Technology Ltd, Saitama, Japan) every day. Mice were exposed to CS daily for 2 h (equivalent to 12 cigarettes per day) for 4 weeks, and treated by periodontitis induction through combining silk suture ligation and *P. gingivalis* infection at the fifth week after the initiation of CS exposure. For inhibition of neutrophils chemotaxis, the CXCR2 inhibitor SB225002 (Selleck, 2 mg/kg) or vehicle control was i.p. administered every other day during the experiment. The study protocol was approved by the Animal Welfare and Ethics Committee of Beijing Chaoyang Hospital (2018-292), and the animal experiments complied with the Regulations on the Administration of Laboratory Animal Affairs in China (2017).

### Lung function test

After anesthesia (1.25% Tribromoethanol, 0.2 mL per 10 g, Nanjing Aibei Biotechnology Co., Ltd) and trachea cannula, mice underwent the pulmonary function analyses using the SCIREQ FlexiVent (SCIREQ, Montreal, Canada) ventilator^60^ according to the manufacture’s instruction. After mice were paralyzed, the lungs were ventilated with a Constant Flow ventilator. Parameters including forced expiratory volume at the end of 0.05 s (FEV0.05), peak expiratory flow (PEF) and expiratory flow at 50% volume (EF50) were collected using the flexiWare software. For each parameter, three measurements were assessed and averaged. Measurements were excluded from analyses if disrupted by a spontaneous breath.

### Neutrophils isolation and culture

Neutrophil isolation was based on protocols that have been previously described.^61^ An equal volume 62%, 75% Percoll density gradient solution was prepared in a centrifuge tube. Fresh blood was diluted 1:1 in serum-free RPMI 1640 without antibiotics, and carefully overlay diluted blood on top of the 62% Percoll. The system was centrifuged at room temperature at 200 × *g* for 25 min and 400 × *g* for 15 min, following by extracting neutrophils layer and the washing twice to obtain neutrophils. A total of 1 × 10^6^ neutrophils were cultured in 1640 complete medium and treated with *P. gingivalis* at 100 multiplicity of infection (MOI) for 24 h.

### Fluorescent in situ hybridization (FISH)

A FAM-conjugated POGI probe (CAATACTCGTATCGCCCGTTATTC) labeled with Spectrum-Green was to detect *P. gingivalis*. A Cy5-conjugated EUB338 universal bacterial probe (GCTGCCTCCCGTAGGAGT) labeled with Spectrum-Red (Thermo Fisher Scientific, USA) was used as the positive control. FISH assay was performed according to the previous instructions.^62^ Microscopic analysis was performed using a Leica Confocal Microscope (Leica Microsystems, Germany).

### RNA extraction and real-time PCR

Total cellular RNA was isolated from cells using TRIzol Reagent (Invitrogen) and reverse transcribed using PrimeScript RT reagent kit (RR047A, Takara) according to the manufacturer’s instructions. Quantitative real-time PCR reaction was performed in triplicates using Rotor-Gene Q Real-time PCR cycler (QIAGEN). The thermal cycles were programmed as follows: 30 s at 95 °C, 40 cycles of 5 s at 95 °C and 34 s at 60 °C. Relative mRNA expression levels for each gene were calculated using the 2^−ΔΔCt^ relative quantification method with *GAPDH* as the endogenous control gene. The designed primer sequences used in the current research are listed in Supplementary Table 1.

### TaqMan qPCR

DNA was extracted using a DNeasy Blood & Tissue Kit (Qiagen, Hilden, Germany) according to the manufacturer’s instructions. Primer sets included two primers designed for *P. gingivalis*, forward (5′-AGCAACCAGCTAC-CGTTTAT-3′) and reverse (5′-GTACCTGTCGGTTTACCATCTT-3′), and one probe (5′-6-FAM-TACCATGTTTCGCAGAAGCCCTGA-TAMRA-3′) synthesized by Invitrogen. Real-time qPCR was performed using TaqMan Universal qPCR 2× Master Mix (Applied Biosystems).

### Immunofluorescence staining and immunohistochemistry

Lung tissues were fixed in 4% paraformaldehyde, dehydrated, embedded in paraffin and sectioned at 4-μm thickness. The tissue sections were mounted on glass slides for immunohistochemical analysis or immunofluorescence staining. After deparaffinized by xylene and ethanol, tissue sections were treated with antigen retrieval EDTA solution (pH = 9.0). Then, the sections were removed endogenous peroxidase activity using 3% H_2_O_2_, sealed with goat nonimmune serum and stained with primary anti-LPS or anti-Ly6G. For immunohistochemistry, the DAB Substrate kit (Proteintech, China) was applied for chromogenic reaction following incubation with the HRP conjugated secondary antibody. For immunofluorescence staining, the primary antibodies were detected with FITC (Proteintech, China) conjugated goat anti-rabbit IgG and then incubated with DAPI to stain the nuclei. The number of Ly6G positive cells was counted by Image-Pro Plus and the percentage of LPS positive staining area was measured by the plugins of IHC Profiler in the ImageJ software. Images were captured on a Leica DM200 LED microscope.

### Enzyme-linked immunosorbent assay (ELISA)

Mouse lung homogenate or MLE-12 cell culture supernatant at logarithmic growth stage after specific treatment were taken as test samples. The sample was diluted to the same concentration with the lowest concentration used as the reference. The levels of TNF-α, IL-1β, G-CSF, CXCL2, MMP-8, and NE in samples were detected by Enzyme-Linked Immunosorbent Assay (ELISA) kits (eBioscience, USA) according to the manufacturer’s instructions. Total protein in the lung tissues was extracted, and the total protein concentration in each lung tissue was determined using the BCA method.

### Neutrophil chemotaxis assay

Chemotaxis assays were performed using 24-well plates with 5-µm pore size inserts (Corning, New York). Trans-well chemotaxis assay was performed in 24-well transwells (6.5-mm diameter, 5-μm pore size; Corning, New York, NY). A total of 5 × 10^5^ per 200 μL neutrophils in serum-free medium were added to the upper chamber, while 600 μL conditional medium (the supernatant of MLE-12 with or without coculture with *P. gingivalis* [MOI] = 1:100 for 24 h) was added to the lower chamber. After 3 h of incubation, neutrophils that had migrated into the lower chamber were collected and counted.

### Flow cytometry

Fresh lung tissues were harvested from mice, incubated in HBSS solution with collagenase type IV and deoxyribonuclease I for 30 min at 37 °C on an incubator shaker to prepare single-cell suspensions. The digested tissues were filtered through 70-μm cell strainers. Then, cells were collected and incubated with specific fluorescent conjugated antibodies of murine cell surfaces markers (anti-CD45 PE-cy7, anti- CD11b- FITC, anti- Ly6G-PerCP cy5.5 from eBioscience). Flow cytometry was performed with BD LSRII, and data were analyzed using Flowjo v.10 software.

### Western blotting

SDS-PAGE and Western blots were carried out according to standard protocols. After proteins transferred to polyvinylidene fluoride (PVDF) microporous membrane (Millipore Corporation, Billerica, MA, USA), specific primary antibodies were used to detect p38 (Proteintech, China), p-p38 (Abcam, UK), p65 (Proteintech, China), p-p65 (Cell Signaling Technology, Danvers, MA, USA), IκBα (Proteintech, China), p- IκBα (Abcam) and β-actin (Proteintech, China). Blots were developed with the enhanced chemiluminescence (ECL) Western blotting detection system (GE Healthcare).

### Statistical analysis

Statistical analysis was performed with a significance level of 0.05 using GraphPad Prism9 software (GraphPad Software, Inc.) and SPSS 25.0. The experimental data were represented as the mean ± standard deviation (SD) of three repeating experiments. Differences analysis was carried out with one-way ANOVA and post hoc Tukey’s or Games-Howell’s multiple comparisons or two-tailed independent Student’s *t* test.

## Supplementary information

Supplementary information

## Data Availability

All data associated with this study are presented in the paper.
