# Supplementary material for: Periodontitis pathogen Porphyromonas gingivalis promotes chronic obstructive pulmonary disease via affecting neutrophils chemotaxis and function
Source: Int J Oral Sci. 2026 Jan 9;18:4. doi: 10.1038/s41368-025-00397-1 (PMC12783702; doi:10.1038/s41368-025-00397-1)
Supplement: Supplementary file 1 — Supplementary information [file 41368_2025_397_MOESM1_ESM.docx]

Supplementary information

**Periodontitis pathogen *Porphyromonas gingivalis* promotes chronic obstructive pulmonary disease via affecting neutrophils chemotaxis and function**

Luyao Zhang^1, †^, Huan Tian^2,3, †^, Yuanyuan Ma^1,4, †^, Jing Xu^1^, Chang Guo^1,5^, Zuomin Wang^3,*^, Jie Ma^1,*^

*† These authors contributed equally to this work.*

*^*^ Corresponding authors.*

**Corresponding authors:**

Jie Ma, MD, Ph.D

Center of Biotherapy, Beijing Hospital, National Center of Gerontology; Institute of Geriatric Medicine, Chinese Academy of Medical Sciences, Beijing, P.R. China

E-mail: majie4685@bjhmoh.cn

Zuomin Wang, MD

Department of Stomatology, Beijing Chaoyang Hospital, Capital Medical University, Beijing, P.R. China

E-mail: wzuomin@sina.cn

**Supplementary Table 1. Primer list.**

| **Gene**  **name** | **Primer Sequences (5’-3’)** | |
| --- | --- | --- |
|  | **Forward primer** | **Reverse primer** |
| GAPDH | AGAAGGTGGTGAAGCAGGCATC | CGAAGGTGGAAGAGTGGGAGTTG |
| IL-1β | CGCAGCAGCACA CAACAAGAGC | TGTCCTCA CCTGGAAGGTCCACG |
| TNF-α | AAAGGGGATTATGGCTCAGG | CTCCCTTTGCAGAACTCAGG |
| MMP-8 | TGCCACGATGGTTGCAGAG | AGGCATTTCCATAATCCCCATTG |
| NE | CCTTGGCAGACTATCCAGCC | GACATGACGAAGTTCCTGGCA |
| CXCL2 | CCAACCACCAGGCTACAGG | GCGTCACACTCAAGCTCTG |
| G-CSF | GCACTATGGTCAGGACGAGAG | GGGGAAATACCCGATAGAGCC |

**
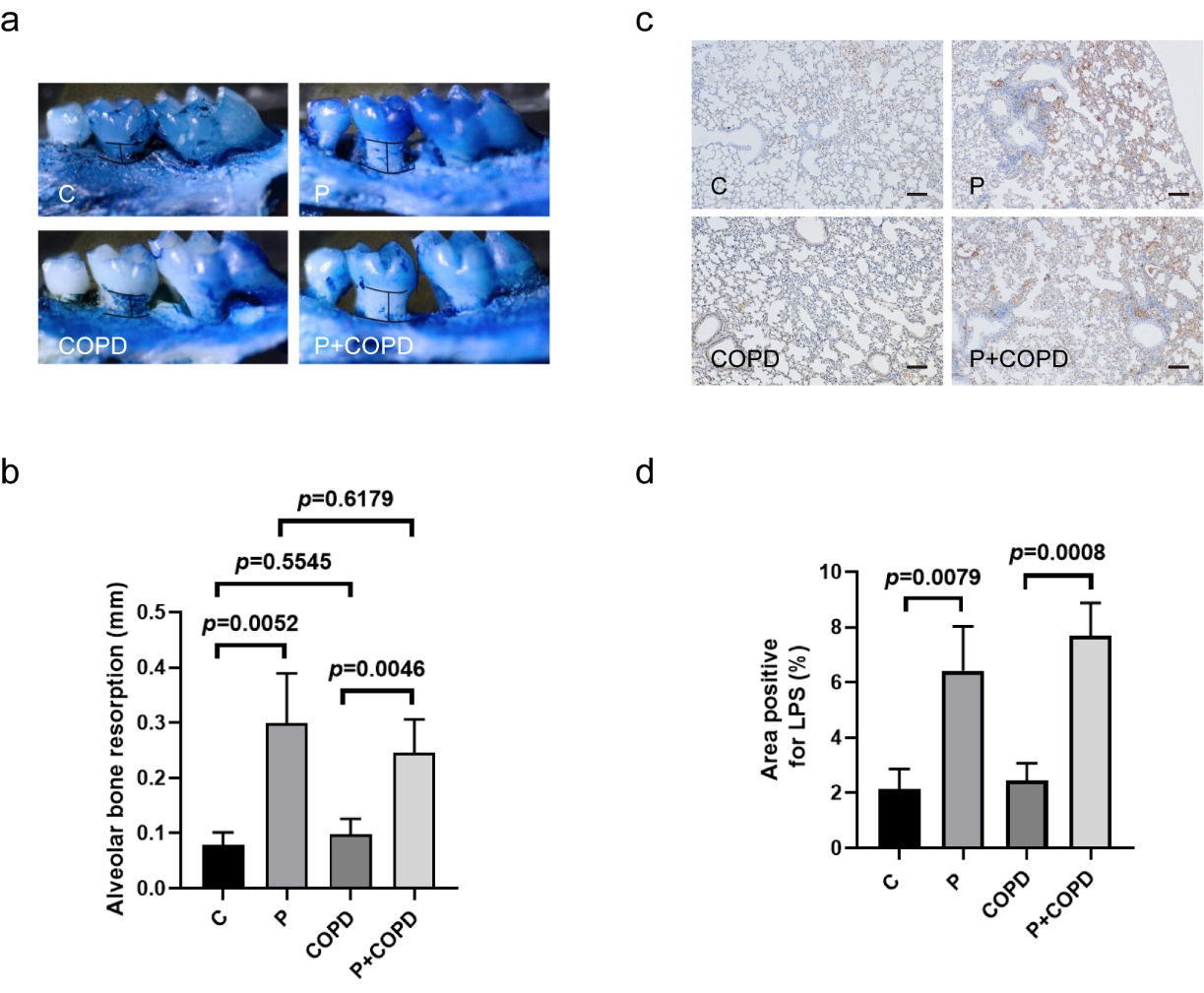
**

**Supplementary Figure 1. Periodontitis-associated pathogen *P. gingivalis* could access to the lung.** (a) Representative images of alveolar bone loss in mouse with control (C group), periodontitis (P group), COPD induced by cigarette smoking (COPD group) and COPD combined with periodontitis (P+COPD group). (b) Alveolar bone resorption in each group (n=6 per group). (c) Representative immunohistochemistry staining image of LPS expression in lung tissue of each group. Scale bars: 100 µm. (d) Quantitative analysis of LPS positive area in (c) (n=5 per group). Significant differences were assessed using one-way ANOVA.

**
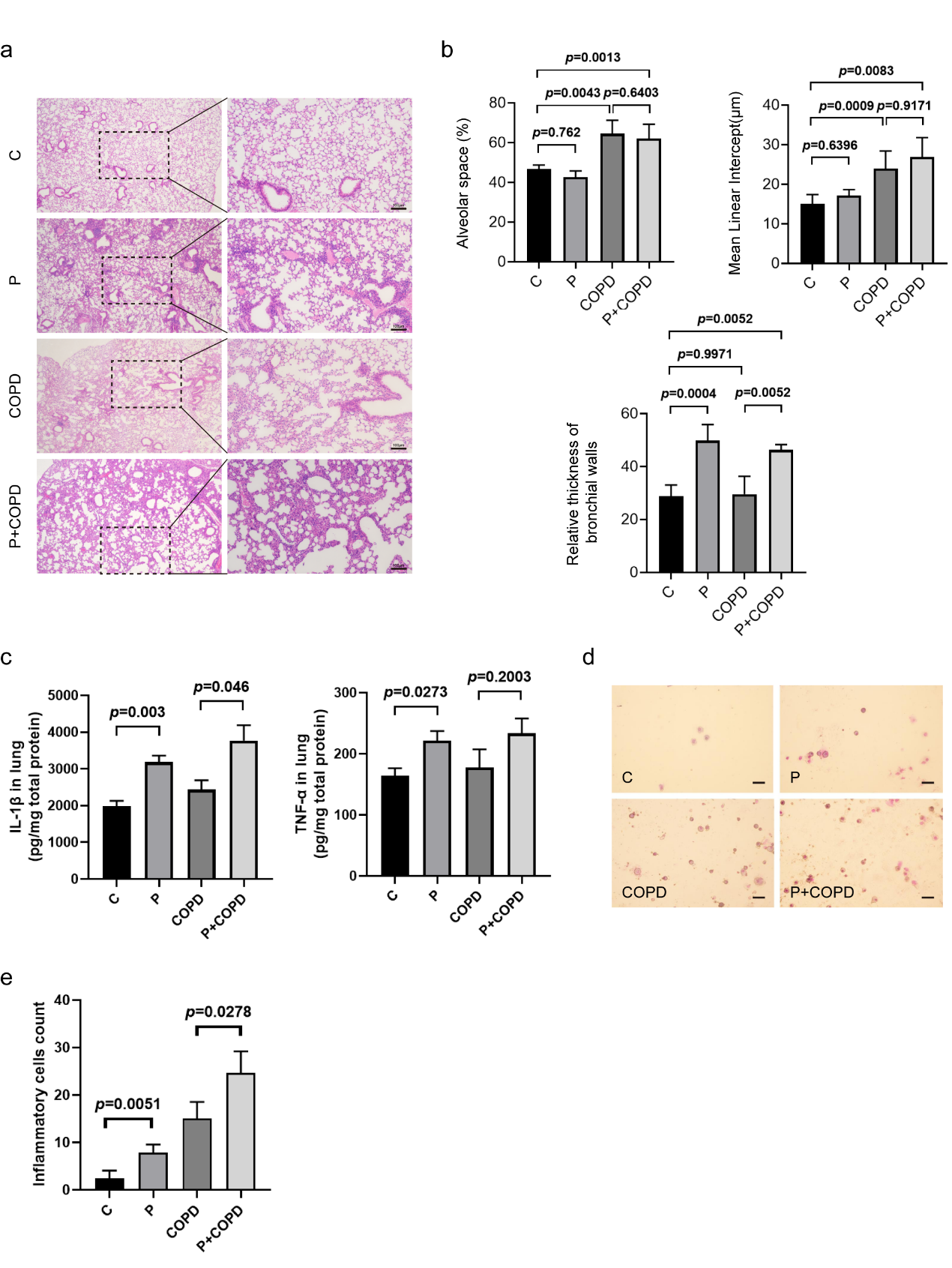
**

**Supplementary Figure 2. Periodontitis aggravated pulmonary inflammatory effect in COPD mice** (a) Representative H&E staining image of lung tissue in each group. (b) Quantitative analysis of alveolar lumen area, relative bronchial wall thickness, and mean linear intercept in lung of each group (n=5 per group). Scale bars: 100 µm. (c) Relative mRNA expression of TNF-α and interleukin IL-1β in lung tissue in each group (n=4 per group). (d) Representative Wright-Giemsa-staining image of bronchial alveolar lavage fluid thin smear in each group. Scale: 50 μm. (e) Quantification of inflammatory cell counting in (d) (n=5 per group). Significant differences were assessed using one-way ANOVA.

**
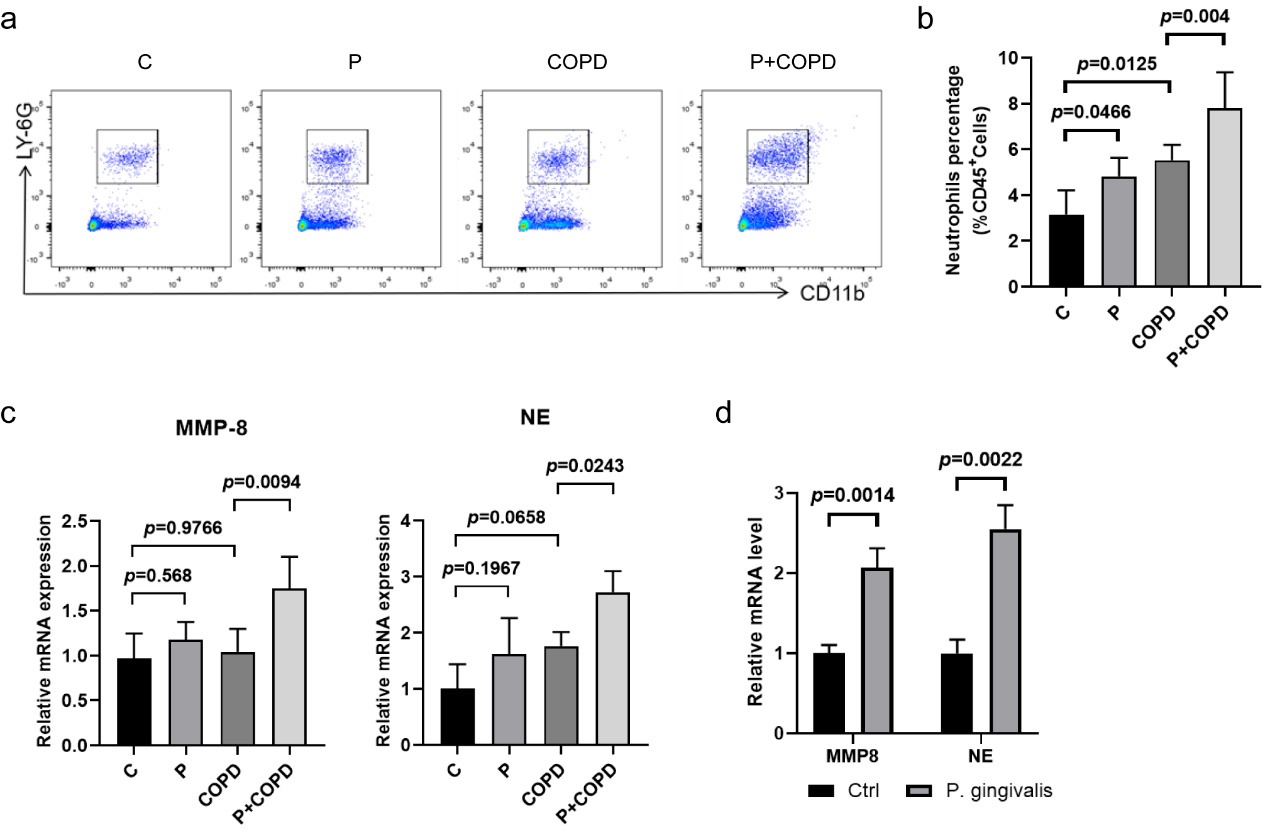
**

**Supplementary Figure 3. *P.* *gingivalis* elevated neutrophils in** **lung tissues and peripheral blood.** (a) Representative plots showing neutrophils level in peripheral blood of each group by flow cytometry. (b) Quantitative analysis of neutrophils level in peripheral blood in (a) (n=6 per group). (c) Relative mRNA expression of matrix metalloproteinases (MMP)-8 and neutrophil elastase (NE) in lung tissue in each group (n=4 per group). (d) 1×10^6^ neutrophils isolated from peripheral blood were treated with *P. gingivalis* at 100 multiplicity of infection (MOI) for 24 h. Relative mRNA expression of MMP-8 and NE in neutrophils (n=3 per group). Significant differences were assessed using two-tailed unpaired Student’s t test and one-way ANOVA.


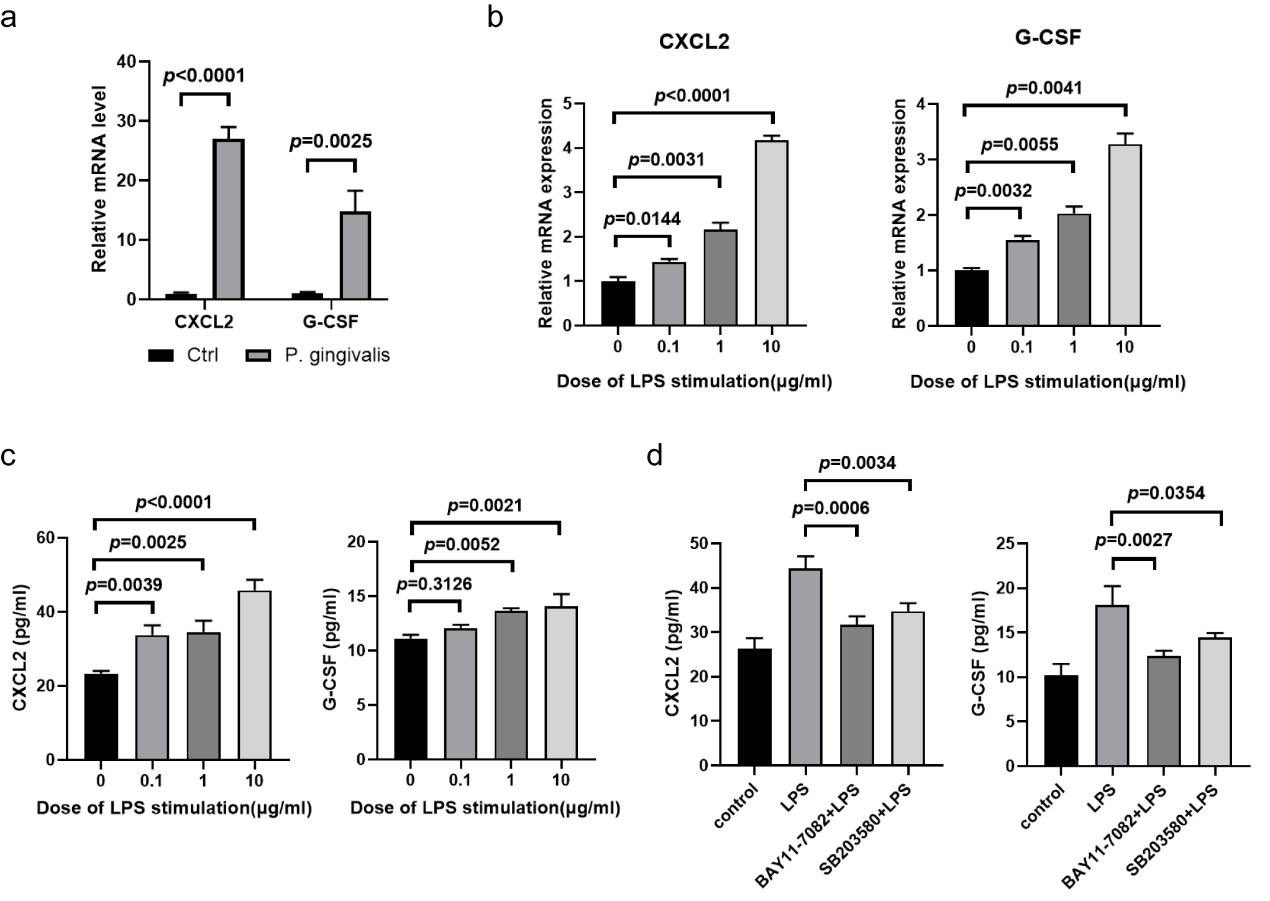


**Supplementary Figure 4. The secretion of CXCL2 and G-CSF from MLE-12 cells were increased under the stimulation of LPS.** (a) Relative mRNA expression of CXCL2 and G-CSF in mouse alveolar epithelial MLE-12 cells treated with *P. gingivalis* [MOI] = 1:100 for 24 h (n=3 per group). (b) Relative mRNA expression of CXCL2 and G-CSF in MLE-12 cells treated with different doses of LPS for 24 h (n=3 per group). (c) The protein expression and secretion of CXCL2 and G-CSF in MLE-12 cells treated with different doses of LPS for 24 h (n=3 per group). (d) The protein expression and secretion of CXCL2 and G-CSF in MLE-12 cells treated with BAY11-7082 and SB203580. Significant differences were assessed using two-tailed unpaired Student’s t test and one-way ANOVA.

**
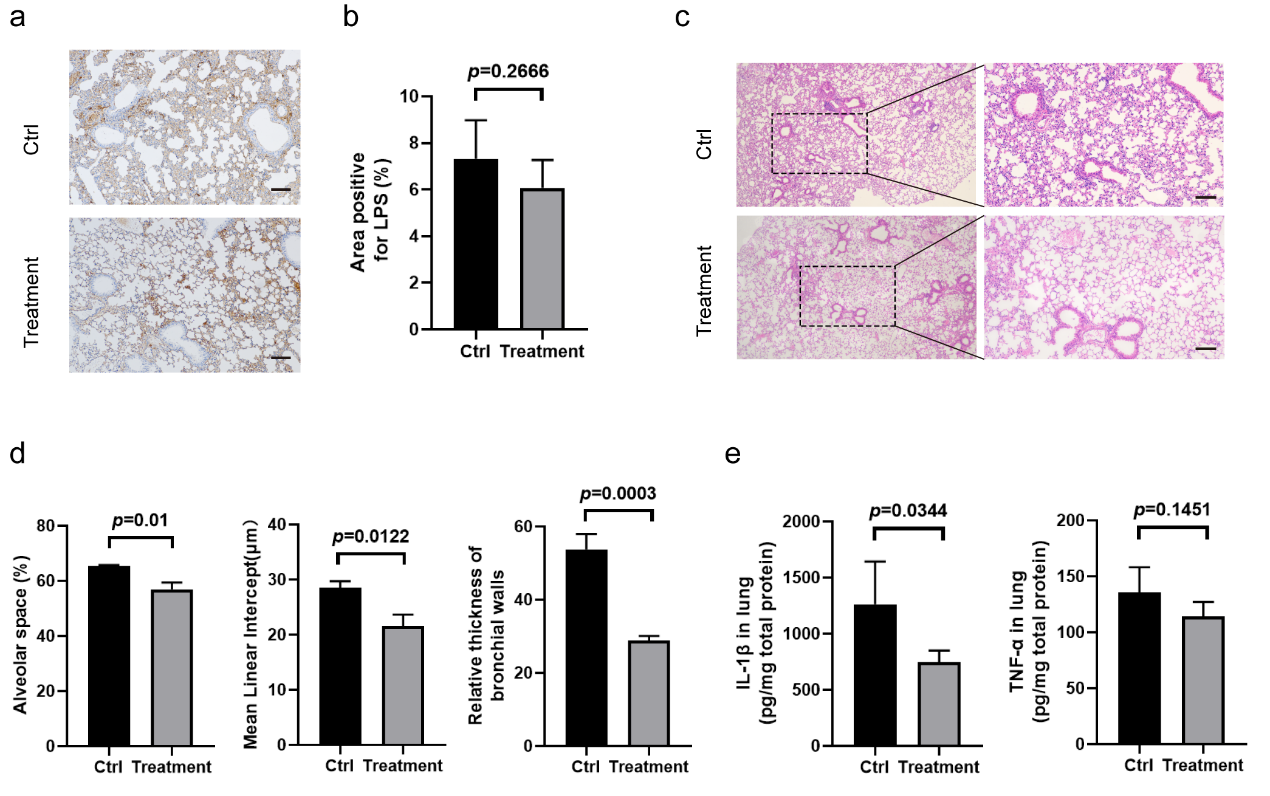
**

**Supplementary Figure 5. Inhibiting neutrophil chemotaxis ameliorated pulmonary inflammation.** (a) Representative immunohistochemistry staining image of LPS expression in lung tissue of mouse with COPD combined with periodontitis (Ctrl), and those treated with CXCR2 antagonist (Treatment). Scale bars: 100 µm. (b) Quantitative analysis of LPS positive area in (a) (n=3 per group). (c) Representative H&E staining image of lung tissue in each group. Scale bars: 100 µm. (d) Quantitative analysis of alveolar lumen area, relative bronchial wall thickness, and mean linear intercept in lung of each group (n=4 per group). (e) The protein expression and secretion of TNF-α and IL-1β in lung tissue of each group was measured by ELISA assay (n=4 per group). Significant differences were assessed using two-tailed unpaired Student’s t test.
